# Precise Characterization of the Penumbra Revealed by MRI: A Modified Photothrombotic Stroke Model Study

**DOI:** 10.1371/journal.pone.0153756

**Published:** 2016-04-19

**Authors:** Cheng Qian, Pei-Cheng Li, Yun Jiao, Hong-Hong Yao, Yu-Chen Chen, Jian Yang, Jie Ding, Xiang-Yu Yang, Gao-Jun Teng

**Affiliations:** 1 Jiangsu Key Laboratory of Molecular and Functional Imaging, Department of Radiology, Zhongda Hospital, Medical School, Southeast University, Nanjing, China; 2 Department of Pharmacology, Medical School, Southeast University, Nanjing, China; Stanford University School of Medicine, UNITED STATES

## Abstract

**Aims:**

To precisely characterize the penumbra by MRI based on a modified photothrombotic stroke mouse model.

**Methods:**

The proximal middle cerebral artery was occluded by a convenient laser system in conjunction with an intravenous injection of Rose Bengal in mice. And the suture MCAO model was performed in seven mice as a comparison of the reproducibility. One hour after occlusion, the penumbra was defined in six random photothrombotic stroke mice by mismatch between perfusion-weighted imaging and the apparent diffusion coefficient map on a home-made workstation. After imaging, three random mice of them were chosen to perform the reperfusion surgery. And the other three mice were sacrificed to stain for several potential penumbra markers, such as c-fos and heart shock protein 90. In the remaining mice, the evolution of the lesions was detected on the apparent diffusion coefficient map, diffusion-weighted imaging and T2-weighted imaging at 1, 3, 6, 12 and 24 hours. After evaluating the neurological deficit scores, the brains were sectioned and stained by triphenyltetrazolium chloride and Nissl.

**Results:**

The mice subjected to photothrombosis showed significant behavioral deficits. One hour after occlusion, the low perfusion areas on the perfusion-weighted imaging interlaced with the hypointense areas on the apparent diffusion coefficient map, demonstrating that the penumbra was located both surrounding and inside the lesions. This phenomenon was subsequently confirmed by the c-fos and heart shock protein 90 staining. The final T2-weighted images of the mice subjected to the reperfusion surgery were also consistent with the penumbra images at one hour. At early stages, the lesions were clearly identified on the apparent diffusion coefficient map; the volumes of the lesions on the diffusion-weighted imaging and T2-weighted imaging did not reach a maximum until 12 hours. The coefficient of variation (CV) of the final lesions in the photothrombotic stroke mice was 21.7% (0.08 of 0.37) on T2-weighted imaging and 27.8% (0.10 of 0.35) on triphenyltetrazolium chloride, representing a high reproducibility (n = 7). While the CV of the lesions in the MCAO stroke mice was only 70% (0.24 of 0.34, n = 4).

**Conclusions:**

This study has provided a precise imaging definition of the penumbra based on a reproducible photothrombotic stroke mouse model.

## Introduction

Stroke is one of the leading causes of serious long-term disabilities and even death around the world. It is estimated that one sixth of people will suffer a stroke at least once in their lifetime [1]. Despite numerous studies during the last decades[2–4], finding potential neuroprotective strategies remains challenging. After a stroke, rescuing the penumbra, a zone of salvageable tissue, has become the major objective of thrombolytic therapy[5]. A distinctive association has been proven between rescuing the penumbra and improvement in outcomes[6, 7].

Imaging provides comprehensive *in vivo* evidence for the evolution of the lesions, the evaluation of hemodynamic issues, and the identification of the penumbra. Appropriate imaging techniques include magnetic resonance imaging (MRI), CT and so on[8, 9]. Among these imaging methods, The noninvasive MRI is currently the most useful and common imaging technique to assess the penumbra. Through the mismatch between perfusion-weighted imaging (PWI) and diffusion-weighted imaging (DWI), it is possible to define the penumbra and then effectively choose candidate patients that are suitable for recanalization therapy in the clinic[10]. However, the traditional penumbra was displayed as surrounding the infarct lesions in most previous studies[11–13], and the evidence of the penumbra was only limited to imaging studies with few pathophysiological-specific markers[14].

To elucidate the pathophysiology of the penumbra, the use of validated animal stroke models is important. Ginsberg et al. discussed that the validity of animal models is self-evident [15]. In fact, there are various advantages in animal models, such as lower cost, reproducibility of lesion size, and suitability for pathophysiological studies. “Close to patient” animal models are needed to bridge the gap between preclinical studies and clinical applications, and to promote precision medicine in the treatment of stroke. The intraluminal suture model, known as the MCAO, is well-recognized in the literature [16]. However, the success rate of occlusion and the reproducibility of lesions are always unsatisfactory with this technique. Furthermore, even with skilled operation, local trauma induced by the surgery is still unavoidable. Unlike the MCAO, the photothrombotic stroke model established by Yao et al. is a pure occlusion method that closely imitates the relevant pathophysiology of thrombosis in the clinic [17]. A highly reproducible lesion could be performed without the need for skilled surgery. However, considering the rats involved in the previous studies, their thick skull mean that a craniotomy is necessary to expose the middle cerebral artery (MCA). Indeed, a distal MCA occlusion leads to the infarct lesions only in the cortex [17], whereas the location of lesions in patients was mostly in the basal ganglia due to the insufficient blood supply [18]. Moreover, the complex laser system used in those studies also limits further popularization.

Therefore, the objective for these experiments was to establish a modified photothrombotic “close to patient” stroke mouse model by occluding the proximal MCA (pMCA) with a convenient laser system, and to provide the precise imaging definition of the penumbra as characterized by early MRI multiparameters.

## Materials and Methods

### Animals

The protocols were approved by the Institutional Animal Care and Use Committee (IACUC), Southeast University (approval ID: SYXK-2010.4987) and in accordance with the Animals (Scientific Procedures) Act, 1986 (UK) (amended 2013). A total of 27 male C57BL/6J mice (22-25g, Yangzhou University, China) were involved and randomly divided into three groups (Photothrombotic stroke group, n = 13; Sham group, n = 7; MCAO group, n = 7). All procedures adhered to the ARRIVE Guidelines for reporting animal research[19]. A checklist is included in S1 Checklist.

### Animal Model

Anesthesia was induced and maintained with the intraperitoneal injection of pentobarbital (60mg/kg; Sigma-Aldrich, USA) during the surgery. The body temperature was monitored continuously and maintained at 37±0.5°C during surgery using a heating pad.

#### 1. Photothrombotic Stroke Model

Focal stroke was induced by occluding the pMCA. Briefly, under a stereoscopic microscope, an incision was made between the right orbit and the external auditory canal. After reflecting the temporalis muscle, the zygomatic arch was snipped. Immediately after the intravenous injection of Rose Bengal (25mg/kg; Sigma-Aldrich, USA) or PBS as a control, illumination was performed on the pMCA for 2 minutes using a 100μm optic fiber connected to a green laser (wavelength 532 nm, 35 mW, GL532TA-100FC, Shanghai Laser & Optics Century, China).

Reperfusion was performed on the occluded pMCA using a 100μm optic fiber connected to an ultraviolet laser (wavelength 355 nm, 16 mW, Shanghai Laser & Optics Century, China) at one hour after occlusion. Afterwards, the temporalis muscle and the skin were closed. The total surgery time duration was approximately 15 minutes. There was no surgery-related mortality.

#### 2. MCAO Model

To evaluate the reproducibility of the photothrombotic stroke model, the most common stroke model, MCAO, was chosen for the comparison. For the MCAO, the surgery was performed as described previously[20]. The total time duration of each operation was at least 45 minutes.

During the surgery of the two stroke models, an ointment was applied to prevent eye dehydration. Lidocaine gel was placed onto the surgical wound after the operation. The mouse was allowed to awaken and then was returned to its cage. Heating pads were used to maintain the cages at a constant temperature of 24°C. The mice were heavily monitored for abnormal health levels.

### Imaging Procedure

MRI was performed on a 7.0 Tesla magnetic resonance scanner (Bruker PharmaScan, Germany). Anesthesia was induced and maintained by inhalation of 1% isoflurane (Shandong Keyuan Pharmaceutical Co., Ltd., China). The body temperature was maintained with a feedback-controlled water bath warming system (MT1025, Bruker Biospin Inc., Germany). The respiratory rate was monitored by a unit (Model 1025, SA Instruments Inc.). Before and after the surgery, magnetic resonance angiography (MRA) was performed to confirm the occlusion by a Flash-three-dimensional sequence with the following parameters: repetition time (TR) /echo time (TE), 15/2.5 ms; field of view (FOV), 20×20 mm; matrix, 256×125. One hour after occlusion, dynamic contrast-enhanced PWI was performed on six random photothrombotic stroke mice by a T2*-weighted EPI sequence with the following parameters: TR/TE, 1,000/9 ms; FOV, 16.5×16 mm; matrix, 128×64; thickness, 1 mm; number of measurements, 200. A bolus injection of Gadolinium (0.3 mmol/kg) through the tail vein was started after the 10% acquisition time. Then, at each time point (1, 3, 6, 12 and 24 hours), T2WI were obtained by a two-dimensional turbo spin-echo sequence with the following parameters: TR/TE, 2,800/50 ms; RARE factor, 8, 3 averages; FOV, 20×20 mm; thickness, 1 mm; matrix, 256×256. DWI (TR/TE, 3,000/30 ms; matrix, 80×64; FOV, 16.5×16 mm) were also obtained with a 2-dimensional spin echo echo-planar sequence. Seven different b-values (0, 100, 200, 400, 600, 800 and 1,000s/mm^2^) were measured to calculate the ADC maps.

### Evaluation of Neurological Deficits

The neurological deficits were evaluated at 24 hours as described previously[21]. The score was evaluated as follows: 0, no deficits; 1, failure to fully stretch the contralateral body and forelimb; 2, circling to the contralateral side; 3, tumbling to the affected side; 4, hardly walking and no automatic action. Each mouse was evaluated by an investigator who was blind to the groups identities.

### Histopathology

At one hour after occlusion, six of the photothrombotic stroke mice were chosen to perform PWI. Then three of them were randomly sacrificed with an overdose of pentobarbital for c-fos (1:1000, sc-52, Santa Cruz, CA) and HSP-90 (1:800, ab13492, Abcam, CA) staining. And another three mice got reperfusion therapy. After the functional evaluation, the remaining mice were also euthanized with an overdose of pentobarbital. The brains were removed and sectioned (1 mm thick; beginning from the olfactory bulb) by a vibratome (version VT1000s, Leica, Germany). Each section was incubated in 1% TTC (AMRESCO LLC) solution for 15 minutes at 37°C. Then, the sections were fixed by 4% formaldehyde for Nissl-staining. The paraffin-blocked tissues were sectioned at a thickness of 4-μm, hydrated in 1% toluidine blue (Sigma-Aldrich, USA) at 50°C for 20 minutes, and then rinsed with double-distilled water. After dehydrating and mounting with Permount, the sections were photographed.

### Image Analysis

The lesions were analyzed by Image J (version 2.1.4.7, NIH). The outlines of the lesions and hemispheric cross sections were traced manually on MR images and TTC sections. To facilitate the comparison between animals and methods, the lesion volumes were corrected for the effects of brain edema by using the following equation: rVL = (VC-VI+VL)/VC[22], where rVL indicates the edema-corrected lesion volumes as a percentage of the contralateral hemispheric volumes; VC and VI indicate contralateral and ipsilateral hemispheric volumes; VL indicates uncorrected lesion volumes. The cerebral blood flow (CBF) map was processed with SPIN software (version 2131, MRI Institute for Biomedical Research). For the penumbra, the areas of low intensity on the ADC map were subtracted from low CBF areas, and then image registration was performed on a home-made data processing workstation.

### Statistical analysis

All statistical data were performed using SPSS software (version 19). All values were expressed as mean ±SD. A general linear model univariate test was applied for global and multiple comparisons between different time points with different MRI sequences. The comparison of lesion volume between T2WI- and TTC-stained sections was made by using the two-tailed paired Student t-test. *p*<0.05 to indicate statistical significance.

## Results

### Establishment of the Photothrombotic pMCA Occlusion Stroke Mouse Model

**Fig 1:**
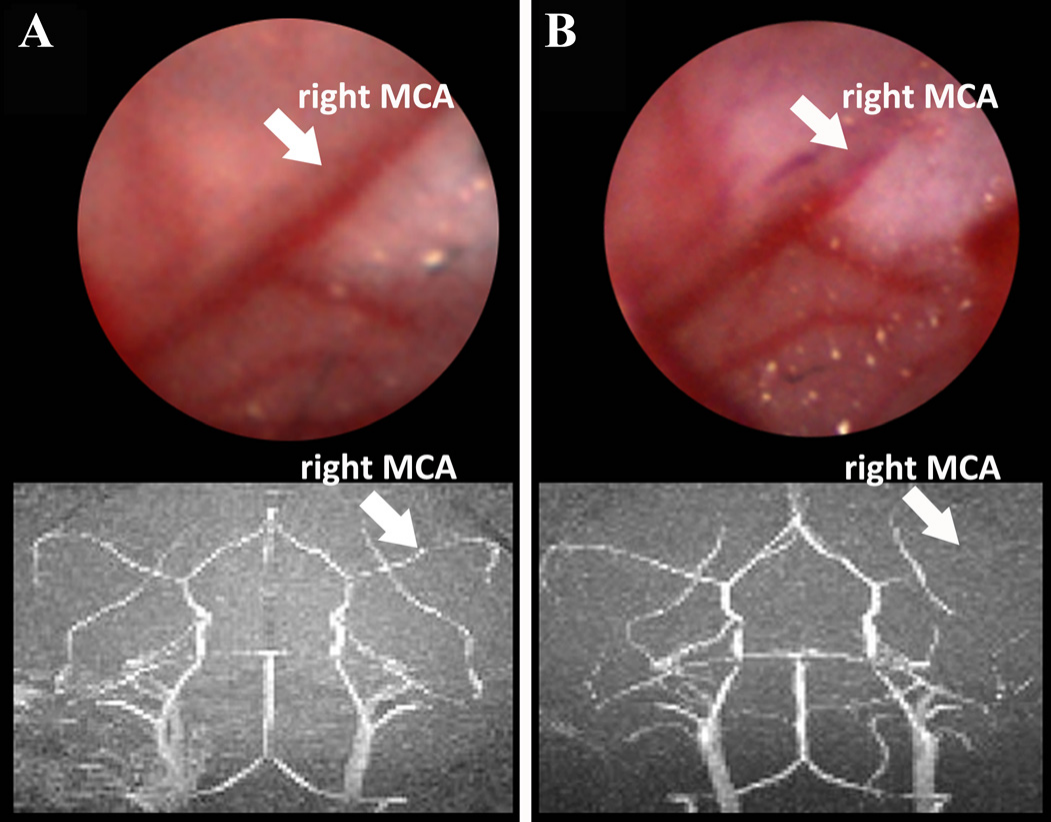
Representative response of proximal MCA to rose bengal-mediated photothrombotic occlusion. A) View of proximal MCA before occlusion under a stereoscopic microscope (upper panel) and on MRA (lower panel). B) View of occluded proximal MCA (white arrows) under a stereoscopic microscope (upper panel) and on MRA (lower panel).

Before the surgery, the intact right MCA could be observed clearly through the transparent skull by a stereoscopic microscope and MRA (Fig 1A). However, it could be difficult to detect after the photothrombotic operation (Fig 1B).

Neurologic deficit scores were used to evaluate each mouse at 24 hours after the occlusion. As shown in Table 1, there were significant behavioral deficits in the photothrombotic stroke group subjected to photothrombosis surgery (n = 7, 2.29±0.76) compared to the sham operation group (n = 7, *p*< 0.05). In detail, the deficit score in six of the seven stroke mice generally ranged from 2 to 3, representing a moderate neurological injury.

**Table 1:** Neurological deficit scores in the photothrombotic stroke and sham groups at 24 hours after MCA occlusion.

Group	number	Neurological deficit scores					Mean±SD
		0	1	2	3	4	
Stroke	7	0	1	3	3	0	2.29±0.76*
Sham	7	7	0	0	0	0	0

*P< 0.05.

### Evolution of the Infarct Volume

**Fig 2:**
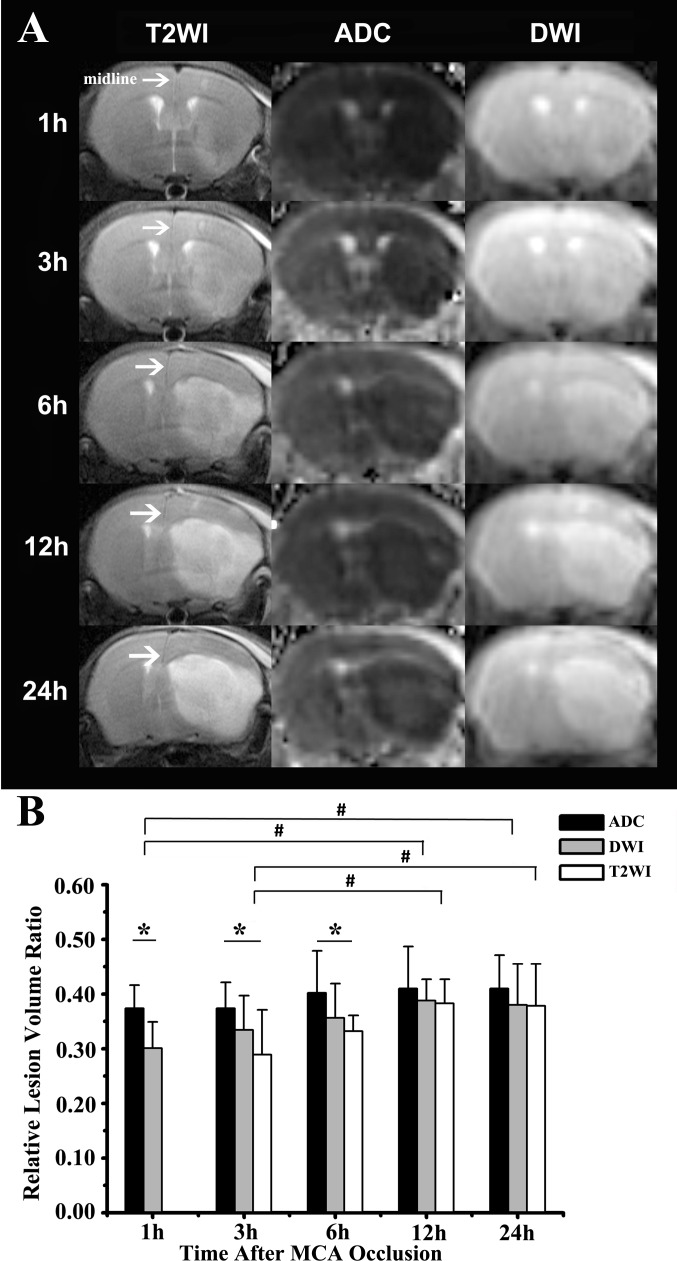
The evolution of the infarct volume on MRI. A) The brain lesions were detected by T2WI, ADC map and DWI at different time points. The curving midlines (white arrows) indicate a severe brain edema. B) The ADC map showed relatively larger lesion volumes at 1 hour (*p<0.05) versus DWI, 3 hours and 6 hours (*p<0.05), and versus T2WI. The relative lesion volumes on DWI and T2WI reached a maximum and matched well with those on ADC map at 12 hours (*p*>0.05), On T2WI, the lesion volumes at 12 and 24 hours were larger (#*p*<0.05) versus that at 3 hours; while on DWI the volumes at 12 and 24 hours were larger (#*p*<0.05) versus that at 1 hour.

The ischemic lesions in the photothrombotic stroke group depicted on different MRI sequences spread from the ventral part of the basal ganglia to the entire basal ganglia and ipsilateral cortex with time. The degree of brain edema became more and more severe, reflected by a shift of the midline (white arrows, Fig 2A). At one hour after MCA occlusion, only the ADC map could display distinct lesions. Indeed, the T2WI detected few significant abnormal signals at that time. At 3 and 6 hours, the ADC map still showed larger relative lesion volumes than T2WI (*p*<0.05). Meanwhile, although there was no significant statistical differences, the rVL on DWI was somewhat smaller than that on the ADC map. The rVL on DWI and T2WI reached a maximum and matched well with that on the ADC map at 12 hours. As shown in Fig 2B, the rVL on DWI at 12 and 24 hours were significantly larger than that at 1 hour, and the rVL on T2WI at 12 and 24 hours were also significantly larger than that at 3 hours (*p*< 0.05). However, the rVL on ADC map at different time points remained consistent (0.42±0.07, 0.37±0.05, 0.40±0.08, 0.41±0.08, 0.41±0.06 at 1,3,6,12,24 hours, respectively, *p*> 0.05).

### Reproducibility of Infarct Volume

No mice died in the photothrombotic stroke group and sham group, while three of the seven mice in the MCAO group died due to large infarct lesions.

**Fig 3:**
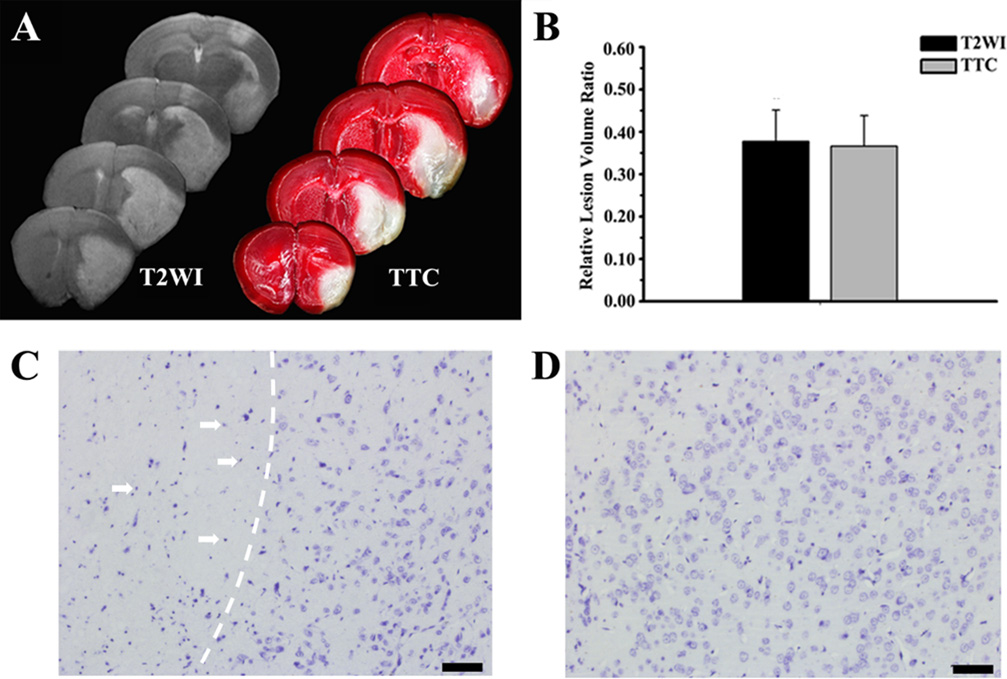
The reproducibility of infarct volume. A) The lesions at 24 hours after occlusion were showed on T2WI (left side) and TTC slices (right side). B) The relative lesion volumes on the TTC-stained slices showed no difference from those on T2WI (*p*>0.05). For Nissl staining, representative images contrasted the ipsilateral (C) and contralateral zone (D). The white arrows indicated that the nucleus of neurons appears with karyolysis and pyknosis in the lesion.

To evaluate the reproducibility of the stroke models, TTC-staining and T2WI were introduced to examine the final relative lesion volumes. The rVL in the photothrombotic stroke group in the TTC-stained slices showed no significant difference with that on T2WI at 24 hours (0.35±0.10 vs 0.37±0.08; *p*> 0.05) (Fig 3A and 3B). The CV of infarct volumes was 21.7% (0.08 of 0.37) on T2WI and 27.8% (0.10 of 0.35) on TTC, suggesting a high reproducibility of this model. The rVL in the MCAO group on T2WI at 24 hours was 0.34±0.24, and the CV of infarct volumes was 70% (0.24 of 0.34) (S1 Fig). Nissl-staining was further used to examine the neurons. The nuclei of neurons in the lesions appeared karyolytic and pyknotic, indicating a regional loss of pigmentation and tissue structure (Fig 3C). Histological abnormalities were not detected microscopically around the lesions or in the contralateral hemisphere (Fig 3D).

### Penumbra on MRI at One Hour

**Fig 4:**
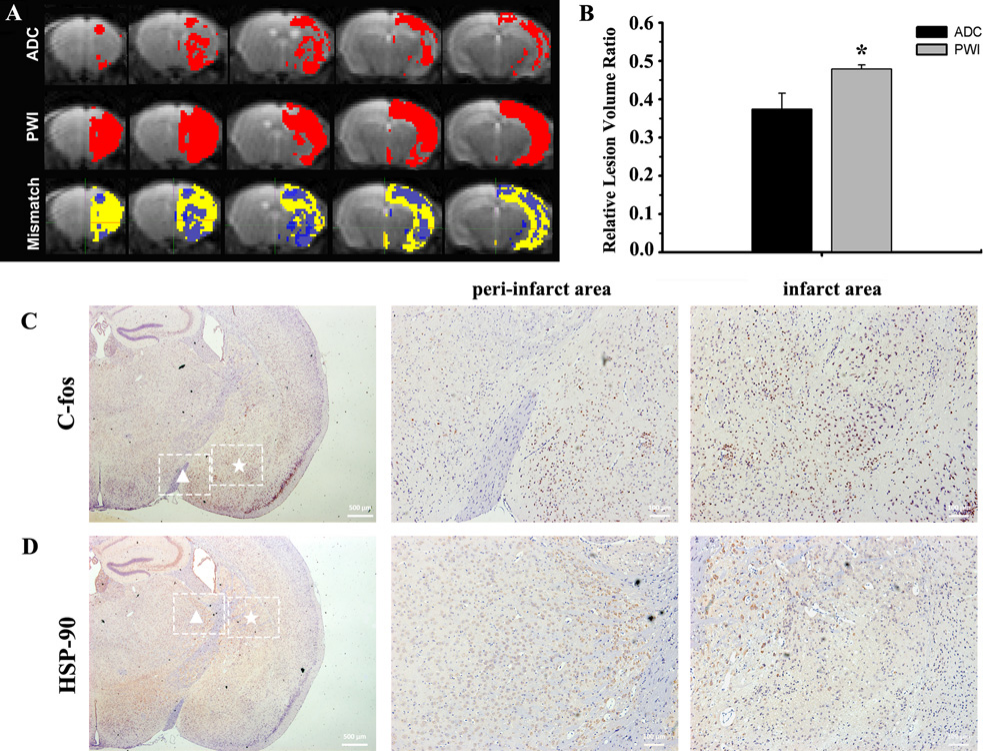
The penumbra at one hour after occlusion on MRI. A) Representative slices from one mouse. Red colored areas represented low perfusion areas on the CBF map or hypointense areas on the ADC map, respectively. On the same map, meanwhile, the light blue colored areas represented the matching areas, which were regarded as the infarct lesions; while the negative mismatch areas were showed in dark blue. And the yellow colored areas represented the mismatching areas, this indicated the penumbra. The penumbra was shown as not only surrounding the lesions but also in the lesions. B) The CBF map on PWI showed considerably larger averaged relative lesion volumes (in percent of ipsilateral hemisphere) (*p<0.05; versus the ADC map). Several potential penumbra markers stains were performed to confirm the location of the penumbra, such as c-fos (C) and HSP-90 (D). The ipsilateral hemispheric slices were taken with a 2.5× objective lens. And the peri-infarct and the infarct areas were taken with a 10× lens indicated by triangle and pentacle, respectively. In both the peri-infarct and infarct areas, c-fos was readily detected, which was also consistent with the HSP-90 staining.

The penumbra was visualized *in vivo* at one hour after occlusion. As the representative slices from one mouse were showed in Fig 4A, red colored areas indicated low perfusion areas on the cerebral blood flow (CBF) map or hypointense areas on the ADC map, respectively. Then, on the same map, the matching areas between the CBF and ADC maps were showed in light blue, while the mismatching areas were showed in yellow, and the negative mismatch areas were showed in dark blue. In detail, the relative volume of low blood flow areas on CBF map appeared considerably larger than the hypointense areas on the ADC map (0.48±0.01vs 0.33±0.08, *p*< 0.05) (Fig 4A and 4B). Interestingly, the mismatch areas, representing the penumbra, could be detected not only surrounding but also in the lesions (Fig 4A). This phenomenon was also confirmed by several potential penumbra markers, c-fos and HSP-90. C-fos was readily observed in both the peri-infarct and infarct areas, which was also consistent with HSP-90 staining (Fig 4C and 4D). The T2-weighted images at 24 hours showed small lesions in the mice subjected to reperfusion, which matched well with the penumbra images at one hour (S2 Fig).

## Discussion

A modified mouse stroke model based on photothrombotic pMCA occlusion has been established in the present study, which is a “close to patient” animal model. There were various methods in the literature for stroke models, such as the electrocoagulation method[23], topical FeCl_3_ application method[24], and MCAO occlusion[25]. The first two methods could cause a certain degree of trauma to the dura mater and parenchyma with craniotomy, leading to spreading depression. For the MCAO method, the surgery is mostly based on the operator’s experience, as the occlusion could be indirectly monitored. Moreover, it is the filament and not the endogenous embolus that occludes the MCA. In the present study, the MCAO model showed a low reproducibility of lesions with high mortality. However, the photothrombotic occlusion is almost limited to the arterial segment with little injury to the surrounding tissues. The principle of photothrombosis is an endothelial damage of the singlet molecular oxygen induced by an endovascular photochemical reaction between the green laser and the photosensitizing solution, resulting in a particularly platelet-based response[26]. This thrombus mostly mimics the clinical thrombosis, making it suitable for a thrombolysis study[27]. The distal MCA was occluded in most previous studies, leading to the lesions being limited to the cortex[28]. In fact, insufficient blood supply makes the basal ganglia more sensitive to ischemia compared to the cortex, and this results in a high incidence rate of infarct in the basal ganglia in clinic[29]. Therefore, large lesions including both cortex and basal ganglia seemed more reasonable by occluding the pMCA in the present study. Using mouse instead of rat could avoid unnecessary trauma induced by the craniotomy. This study also introduced a convenient and cheap laser system with an optical fiber instead of the complex beam steerage in previous studies. This system shortened the irradiation time to just 2 minutes, and avoided more irradiation trauma. There were several advantages in this modified stroke model: 1) The surgery operation could be finished rapidly within 15 minutes. 2) No professional surgical techniques were required. 3) Additional trauma induced by craniotomy could be avoided. 4) The final lesions involved both cortex and basal ganglia, resulting in moderate deficit scores with low mortality. 5) A high reproducibility of infract volumes could be achieved. 6) A convenient and cheap laser system was introduced. Such advantages should facilitate its further study in various fields.

In the present study, noninvasive MRI was introduced to characterize, from early stages and onwards, the evolution of lesions in real-time. Compared with the other sequences (T2WI and DWI), the ADC map indicated earlier and clearer lesions, and it has been proved to predict the final lesions[30]. Combined with the DWI or ADC map, the CBF map could discern the penumbra [31]. The penumbra has become the major objective of the thrombolytic therapy. However, the therapy time window for penumbral salvage is within a few hours of stroke onset. Patients with a penumbra, if detected in time, may benefit from the thrombolytic therapy[32]. There have been several animal studies on the penumbra[33, 34]. For example, several studies were undertaken on another photothrombotic “ring” stroke model[35]. Directly irradiating the cortex with a ring filter resulted in a central area surrounded by the lesions. However, the animal model lesions were present within the simultaneous vasogenic edema and cytotoxic edema, which is not similar with the stroke in human beings. To our knowledge, we are the first to precisely make the penumbra visualization in this model. Different from the conventional concept of the penumbra[34–36], an interesting finding in the present study was that the low perfusion areas on PWI interlaced with the hypointense areas on the ADC map. This resulted in the penumbra not only surrounding but also being found in the lesions (Fig 2A). The reason for this phenomenon might be the complicated anatomy of the arteries. And the residual collateral circulation may also supply blood to a number of small regions in the lesions, leading to several salvageable tissues. Coincidentally, this phenomenon was also detected in the acute stroke patients based on our home-made data processing workstation (unpublished results). The precise visualization of the penumbra seemed to accord with the complex pathophysiology in human beings. It has been reported that the expression of c-fos was localized in the penumbra and the normal regions adjacent to the lesions[37]. Hsp90 was already reported to play important roles in the regulation of cellular homeostasis and stress response, and it has shown a potential relationship with the penumbra[38]. Our study further demonstrated the relationship between these pathobiological markers and the penumbra. However, it is still unclear if these specific biological markers could represent the penumbra[39, 40]. Therefore, better understanding of the penumbra both on imaging and pathophysiology could be received based on our study, which may promote research in stroke and anti-stroke agents.

There were some limitations to note. Due to several small vessels around the zygomatic arch, particular attention should be paid to avoid hemorrhage during the process of surgery. This issue could be avoided with enough practice and carefulness. To shorten the scan time of PWI, the images were acquired without high-resolution. Shimming before scanning and adjusting the scanning parameters would improve image resolution.

In conclusion, we have established a modified reproducible stroke mouse model by photothrombotic pMCA occlusion with a convenient laser system. On MRI, the precise location of the penumbra in our study was considered to mimic the complex situation in human beings. Furthermore, using this model could improve the interpretation of the results, providing promising functional relevance for application in clinical scenarios.

## Supporting Information

S1 Checklist Completed ‘‘The ARRIVE Guidelines Checklist” for reporting animal data in this manuscript. (PDF)

S1 Dataset The evolution of lesion volume for all endpoints. (XLSX)

S2 Dataset The final lesion volumes on TTC and T2WI. (XLSX)

S3 Dataset Lesion volumes on PWI and DWI. (XLSX)

S1 Fig The reproducibility of infarct volume on MCAO model. A) The same slices from different mice at 24 hours were showed on T2WI. B) The relative lesion volumes of each mouse subjected to MCAO.
(TIF)

S2 Fig Effect of reperfusion on the penumbra. A) Slices of one representative mouse only subjected to photothrombotic occlusion on T2-weighted images at 24 hours. B) The T2-weighted images of one representative mouse subjected to reperfusion at 24 hours. C) Slices of the same mice subjected to reperfusion on the PWI/DWI mismatch images at one hour.
(TIF)
