# Supplementary figures and images for: Precise Characterization of the Penumbra Revealed by MRI: A Modified Photothrombotic Stroke Model Study
Source: PLoS One. 2016 Apr 19;11(4):e0153756. doi: 10.1371/journal.pone.0153756 (PMC4836676; doi:10.1371/journal.pone.0153756)

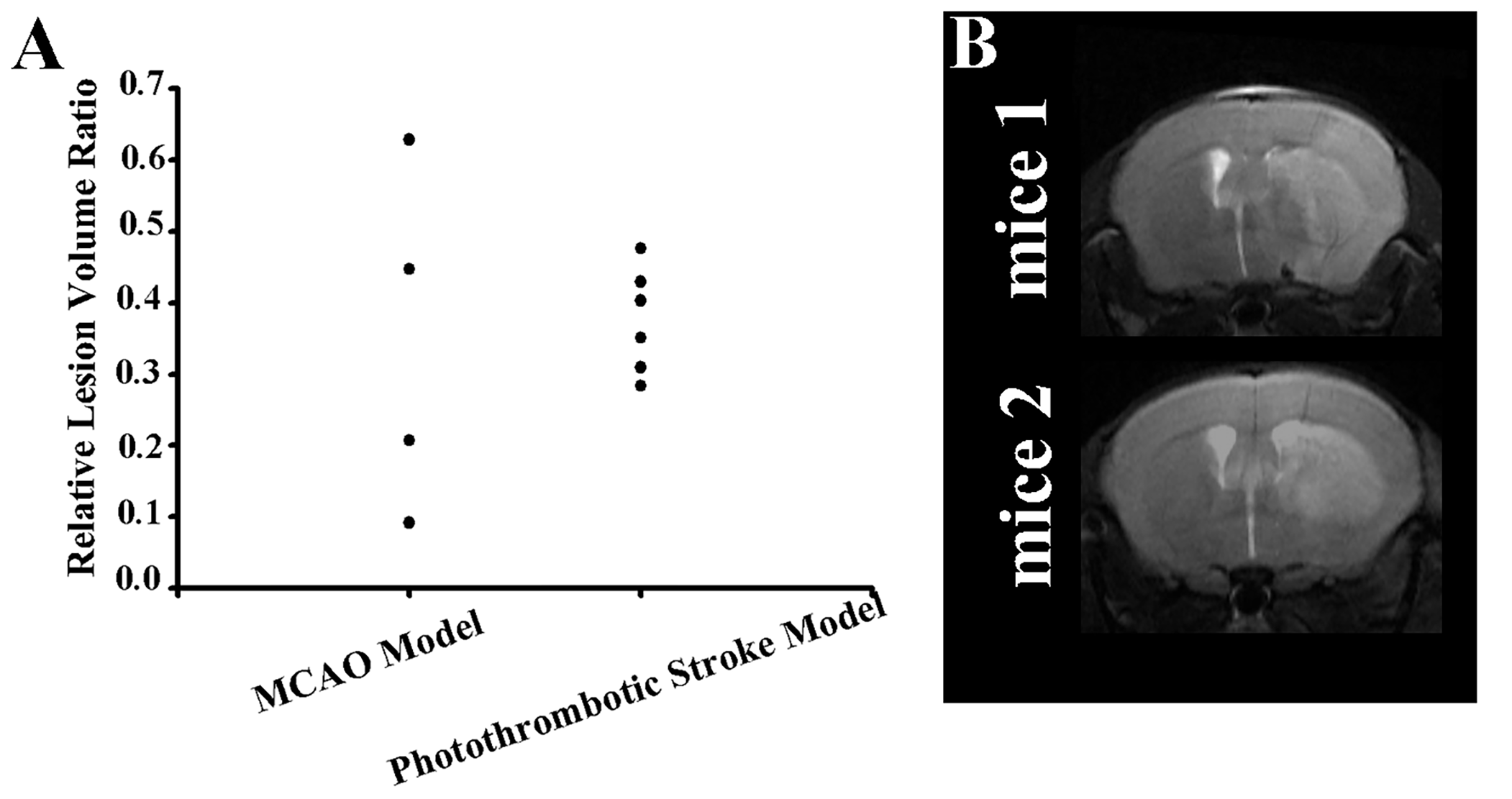

Supplement: S1 Fig — A) The same slices from different mice at 24 hours were showed on T2WI. B) The relative lesion volumes of each mouse subjected to MCAO. (TIF) [file pone.0153756.s005.tif]

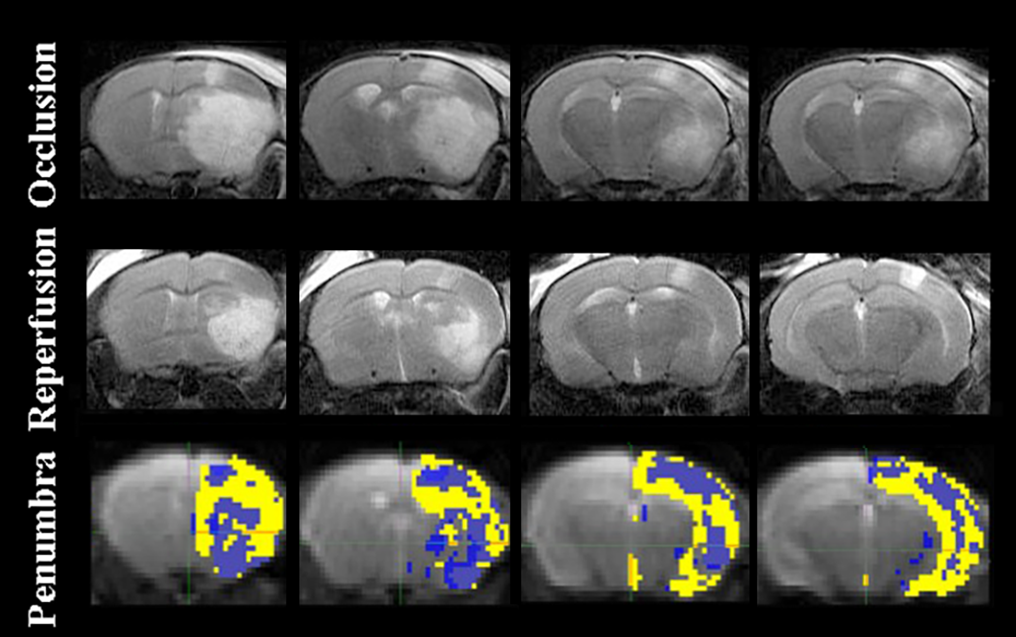

Supplement: S2 Fig — A) Slices of one representative mouse only subjected to photothrombotic occlusion on T2-weighted images at 24 hours. B) The T2-weighted images of one representative mouse subjected to reperfusion at 24 hours. C) Slices of the same mice subjected to reperfusion on the PWI/DWI mismatch images at one hour. (TIF) [file pone.0153756.s006.tif]
